# Contribution of pulmonary function tests (PFTs) to the diagnosis and follow up of connective tissue diseases

**DOI:** 10.1186/s40248-019-0179-2

**Published:** 2019-05-15

**Authors:** Nicola Ciancio, Mauro Pavone, Sebastiano Emanuele Torrisi, Ada Vancheri, Domenico Sambataro, Stefano Palmucci, Carlo Vancheri, Fabiano Di Marco, Gianluca Sambataro

**Affiliations:** 1 Regional Referral Center for Rare Lung Diseases, A. O. U. “Policlinico-Vittorio Emanuele” Department of Clinical and Experimental Medicine, University of Catania, Catania, Italy; 2 Respiratory Physiopathology Group. Società Italiana di Pneumologia. Italian Respiratory Society (SIP/IRS), Milan, Italy; 3 Artroreuma S.R.L. Outpatient Clinic accredited with the Italian National Health System, Corso S. Vito 53, 95030 Mascalucia (CT), Italy; 4 Department of Medical Surgical Sciences and Advanced Technologies- Radiology I Unit, University Hospital “Policlinico-Vittorio Emanuele”, Catania, Italy; 5 Department of Health Sciences, Università degli studi di Milano, Head Respiratory Unit, Papa Giovanni XXIII Hospital, Bergamo, Italy

**Keywords:** Interstitial lung disease, Rheumatoid arthritis, Connective tissue disease, Antisynthetase Syndrome, Systemic sclerosis, Sjӧgren Syndrome, Dermatomyositis, Polymyositis, Mixed connective tissue disease, Interstitial pneumonia with autoimmune features

## Abstract

**Introduction:**

Connective Tissue Diseases (CTDs) are systemic autoimmune conditions characterized by frequent lung involvement. This usually takes the form of Interstitial Lung Disease (ILD), but Obstructive Lung Disease (OLD) and Pulmonary Artery Hypertension (PAH) can also occur. Lung involvement is often severe, representing the first cause of death in CTD. The aim of this study is to highlight the role of Pulmonary Function Tests (PFTs) in the diagnosis and follow up of CTD patients.

**Main body:**

Rheumatoid Arthritis (RA) showed mainly an ILD with a Usual Interstitial Pneumonia (UIP) pattern in High-Resolution Chest Tomography (HRCT). PFTs are able to highlight a RA-ILD before its clinical onset and to drive follow up of patients with Forced Vital Capacity (FVC) and Carbon Monoxide Diffusing Capacity (DL_CO_). In the course of Scleroderma Spectrum Disorders (SSDs) and Idiopathic Inflammatory Myopathies (IIMs), DL_CO_ appears to be more sensitive than FVC in highlighting an ILD, but it can be compromised by the presence of PAH. A restrictive respiratory pattern can be present in IIMs and Systemic Lupus Erythematosus due to the inflammatory involvement of respiratory muscles, the presence of fatigue or diaphragm distress.

**Conclusions:**

The lung should be carefully studied during CTDs. PFTs can represent an important prognostic tool for diagnosis and follow up of RA-ILD, but, on their own, lack sufficient specificity or sensitivity to describe lung involvement in SSDs and IIMs. Several composite indexes potentially able to describe the evolution of lung damage and response to treatment in SSDs are under investigation. Considering the potential severity of these conditions, an HRCT jointly with PFTs should be performed in all new diagnoses of SSDs and IIMs. Moreover, follow up PFTs should be interpreted in the light of the risk factor for respiratory disease related to each disease.

## Background

Connective Tissue Diseases (CTDs) are often characterized by pulmonary involvement. The main clinical presentation is Interstitial Lung Disease (ILD), characterized by an involvement of the lung interstitium with inflammatory cells and/or exaggerated deposition of extracellular matrix by myofibroblasts. Pulmonary Artery Hypertension (PAH, a potentially lethal condition characterized by high blood pressure in lung arteries) and Obstructive Lung Diseases (OLDs, an airway obstruction with airflow limitation) can also occur. These three conditions, with several pathogenic pathways directly or indirectly related to CTDs can be present alone or in combination. The lung disease can be severe, and often represents the main cause of death for these patients [1]. Currently, Pulmonary Function Tests (PFTs) in clinical practice appear to be confined to the follow up of autoimmune patients in which an established lung involvement is known. However, patients can remain asymptomatic for lung time despite evidence of lung involvement already being present (“Velcro” crackles in auscultation, impaired PFTs or evidence of lung involvement in chest imaging**)** [2, 3]. In view of this, PFTs can also represent a useful, inexpensive and safe tool for the diagnosis of lung disease in CTDs and its management in the early stages, in which the disease could be more responsive to treatment.

The aim of this paper is to review lung involvement in CTD and highlight the role of PFTs in its diagnosis and follow up.

### Rheumatoid arthritis

Rheumatoid Arthritis (RA) is an autoimmune, potentially systemic disease characterized by an inflammatory, symmetric, additive and erosive arthritis affecting about 1% of the population of developed countries [4]. The pathogenesis of the disease is currently not fully understood, but the lung seems to be a possible source of autoimmunity. Exposure to dust and smoke can lead to the citrullination of peptides, provoking an autoimmune response, with the production of Anti Cyclic Citrullinated Antibodies (ACPA) which represent one of the most specific items for the diagnosis [5, 6]. Probably due to autoimmune mechanisms, RA can be directly associated with respiratory system damage not only with ILD, but also with bronchiectasis, pleural effusion, rheumatoid parenchymal nodules and, rarely, vascular disease. Indirectly RA-associated respiratory damage (generally with ILD or respiratory tract infections) can be caused by the drugs used for treatment [7].

RA-ILD occurs in 4–68% of patients depending on the criteria used for identification, while 10% of RA patients showed clinically relevant ILD [8, 9]. The most common pattern in High-Resolution Chest Tomography (HRCT) or Surgical Lung Biopsy (SLB) is Usual Interstitial Pneumonia (UIP), followed by Nonspecific Interstitial Pneumonia (NSIP) and rarely other patterns [7]. Risk factors linked to RA-ILD are age, male sex, smoking history, positivity for ACPA, longstanding and/or persistently active disease, presence of rheumatoid nodules and articular erosions, and genetic predisposition with several Human Leukocytes Antigens (HLAs) [10, 11]. A worse prognosis is reported in UIP pattern rather than NSIP or Organized Pneumonia (OP), but currently there is no consensus in the literature on whether a specific HRCT pattern in RA-ILD has a similar or better prognosis compared to the same pattern in Idiopathic Interstitial Pneumonia (IIP) or Idiopathic Pulmonary Fibrosis (IPF) [10]. Female sex and higher levels of Carbon Monoxide Diffusing Capacity (DL_CO_) were associated with a better prognosis [12].

RA patients can also develop OLD, mainly Chronic Obstructive Pulmonary Disease (COPD), Bronchiectasis (BR) and bronchiolitis associated with ILD. COPD is obviously the most frequent OLD in RA [13]. A history of smoking plays a crucial role, but regardless of this, RA showed a pooled risk ratio of OLD of 1.99 even in non-smoker patients [14], with a mortality comparable to RA-ILD [15].

Finally, RA was also associated with Combined Pulmonary Fibrosis and Emphysema (CPFE), a syndrome often characterized by severe dyspnoea, preserved lung volumes and severe reduction of DL_CO_, with emphysema localized in the upper lobes and ILD in the lower ones and possible development of PAH [16]. Initially described in patients with a history of smoking, it was described in 27% of RA-ILD patients who had never smoked and it was independently associated with higher mortality [17]. PFTs appear to be able to predict the evolution of RA-ILD. Lower Forced Vital Capacity (FVC) and DL_CO_ and their decline over 6 months are associated with a severe disease [18], while DL_CO_ ≤54% was identified as a cut-off for RA-ILD progression with sensitivity of 80% and specificity of 93% [19]. Another interesting study correlated mortality with a FVC lower than 61.8% of that predicted at the baseline or a decline of 10% from the baseline in any time [20]. In the same study, adjusting data for age, sex and smoking, a specific HRCT pattern was not associated with survival. Instead, the six-minute walk test (6MWT) during RA-ILD was not studied in depth, probably due to possible impairment caused by articular disease.

Despite the fact that pulmonary involvement is the most common extra-articular manifestation of RA with an impact on prognosis, guidelines for the management of lung involvement during RA are not currently available. Generally, RA patients were studied for lung involvement at the onset of suggestive symptoms. However, as already mentioned, several ILDs are able to produce a decline in PFTs in patients who were initially asymptomatic [2, 3]. Moreover, a UIP pattern in RA-ILD is associated with a longer disease duration, leading some authors to theorise a progression from a NSIP pattern [12]. In view of this concept, it could be useful to determine ILD before structural damage, in order to tailor appropriate treatment. Indeed, increasing evidence suggests the use of anti-fibrotic drugs for the treatment of RA-ILD [21] and it appears reasonable to suppose that this could be a new indication for these drugs in the future.

The recognition of lung involvement in RA can also be useful in the management of the disease. In fact, several treatments for RA were associated with the development or the exacerbation of a pre-existing ILD, mainly Methotrexate and TNFα blockers [22, 23]. Patients with RA-ILD may benefit from the use of Tocilizumab and Abatacept, two biologic drugs to date not associated with fibrotic lung involvement [24]. Moreover, the early recognition of OLD could allow the elimination of modifiable risk factors, and drive the treatment towards the use of drugs with a lower risk of pulmonary infections [25].

In our opinion, a physical examination looking for “Velcro” crackles and PFTs should be performed in all new diagnoses of RA, and repeated at least every 12 months. HRCT should be reserved for RA patients with a positive physical examination, impaired PFTs at baseline or reduction of 10 and 15% respectively for FVC and DL_CO_ at 6–12 months, according to international guidelines for IIPs [26].

In the event that the clinical picture suggests CPFE, the issue of preserved lung volumes would mean that the only screening possible for these patients would be based on a clinical examination of their reduced DL_CO_, which could then be studied in depth by HRCT and echocardiogram.

### Scleroderma spectrum disorders

The most important disease considered in Scleroderma Spectrum Disorders (SSDs) is Systemic Sclerosis (SSc). The disease affects about 1 out of 10,000 persons [27] and is characterized by fibrosis of the skin and internal organs, Raynaud Phenomenon (RP) and vasculopathy. SSc is classically subdivided into 4 expressions according to skin involvement: Diffuse SSc (dSSc), Limited SSc (lSSc), SSc sine scleroderma (ssSSc) and Overlap Syndromes (OS), in which classic sclerodermic signs are associated with features typical of other CTDs [28]. SSc is the most severe CTD and lung involvement is the major cause of death both for ILD and PAH [29]. Serological positivity can serve as a marker of lung involvement: Scl70 positivity is associated with ILD, while Anticentromeric antibodies (ACA), anti RNA polymerase I, II and III (above all when associated with U1RNP positivity) are associated with PAH and a worse prognosis. Moreover, Th/To antibodies are associated with ILD preceding PAH, mainly in patients with ssSSc [30].

Scleroderma-related ILD is mainly characterized by an NSIP pattern on HRCT, followed by UIP. ILD affects about 80% of SSc patients, but only 25–30% will develop progressive disease within 5 years of the disease onset (counted from the first non-RP scleroderma sign) that will stabilise into structural damage in 4–6 years [28]. It can be classified into limited and diffuse ILD according to Goh’s staging system, correlating with a worse prognosis for a disease extent threshold ≥20% [31].

On the other hand, PAH affects about 15% of SSc patients, with a worse prognosis than the idiopathic form [29]. SSc-PAH can arise from different pathogenic mechanisms: caused by disease of the small pulmonary arteries (group 1 PAH, mainly in lSSc patients), due to myocardial fibrosis (rarely, group 2 PAH), or secondary to ILD (group 3 PAH, mainly in dSSc patients) [32].

Finally, OLD can also be found in in SSc patients. CPFE was described in 12.3% of patients, a few of whom were non-smokers [33].

Unfortunately, the use of PFTs in SSc appears to be less useful than in RA-ILD or IPF. This is understandable, taking into account that SSc has different pathogenic mechanisms and generally a different HRCT pattern than RA and IPF. In fact, unlike IPF, the use of FVC as a good predictor of lung ILD severity is not sufficiently supported, despite its wide use as a primary outcome in clinical trials. Five validation studies were undertaken to study the best PFT items, but in a recent meta-analysis no items from PFTs showed good sensitivity or specificity for the early diagnosis of ILD [34]. Therefore, an HRCT should be performed in all new diagnoses of SSc, especially for dSSc.

Also in follow up, ILD extension was associated with FVC and DL_CO_ only at the baseline, but not with their decline [35]. FVC showed high variability among SSc patients, with even amelioration from low baseline levels: a fast decline was experienced mainly in patients with early disease [36]. In contrast, using baseline data from Scleroderma Lung Study I and II, DL_CO_ provides the best estimate of ILD measured with HRCT quantification [37], at least in patients without PAH [38]. DL_CO_ is useful not only in the prediction of disease severity, but also in the early forecast of organ damage, in the estimation of disease activity (DA) and correlates with DA index in Nailfold Videocapillaroscopy (NVC) [39–42]. Obviously, DL_CO_ can be reduced by the presence of PAH [43]. This data reduces the specificity of DL_CO_ in the description of SSc-ILD and should be taken into account in its interpretation. Finally, Total Lung Capacity (TLC) also showed good correlationas a measure of ILD severity, but needs to be studied more extensively [31]. It is likely that, as suggested by Caron et al. in their interesting meta-analysis [34], a composite index could better describe SSc-ILD extension and severity than the use of a single item alone. The Outcome Measures in Rheumatology (OMERACT) proposed a decline of FVC ≥ 10% or a reduction of ≥5 to < 10% with a decline of DL_CO_ ≥15% to define ILD progression, but these measures need to be validated [44].

A similar point can be made for the detection of PAH. The diagnosis should be confirmed with Right Heart Catheterization (RHC), but several PFT items can be used for screening. An isolated reduction of DL_CO_ ≤55% without significant ILD and an FVC/DLCO ratio > 1.6 were found to be useful in forecasting future PAH in patients with SSc [45]. Unfortunately, DL_CO_ can be biased by frequently occurring concomitant ILD that can contribute to its reduction. A subdivision of DL_CO_ into two transfer components (membrane conductance for CO and alveolar capillary blood volume) has been proposed as an ideal solution. This partition can be calculated combining DL_CO_ with the transfer factor of the lung for nitric oxide (TLNO) [46]. However, a large study reported that the combined measurement of DL_CO_ and TLNO does not improve the recognition of SSc-PAH [47]. Therefore, currently the most sensitive method to screen SSc patients for PAH is a composite, two-step, algorithm named DETECT. Items considered in this algorithm are: FVC/DL_CO_ ratio, presence of ACA and telangiectasias, serum level of NT-proBNP and urate, presence of right axis deviation on Electrocardiogram, right atrium area and tricuspid velocity in echocardiography. DETECT showed better performance than the International European Society of Cardiology/European Respiratory Society guidelines in the diagnosis of SSc-PAH [48].

Mixed Connective Tissue Disease (MCTD) could be considered in SSDs. This autoimmune disease is characterized by the presence of the U1-RNP antibody and clinical features of SSc, Systemic Lupus Erythematosus (SLE) and Polymyositis/Dermatomyositis (PM/DM) [49]. The annual incidence of MCTD is about 1.9 per 100,000 population, with a mortality similar to general population [49]. It is still a matter of debate whether MCTD should be considered a separate entity or a form of Undifferentiated Connective Tissue Disease (UCTD)/OS, considering that the clinical picture for MCTD usually becomes clear within 3.6 years, but the rate of differentiation in other CTDs in 10 years of follow up is low (8.5% for SLE and 6.3% for SSc) [49].

Pulmonary involvement, particularly PAH, is the major cause of death in MCTD patients [50]. ILD in MCTD usually shows an NSIP pattern, followed by UIP and it occurs in about 50% of patients [51]. Out of these, 19% show a severe, rapidly progressive ILD, with a mortality of 20% in a follow up of 4.2 years [52]. On the other hand, PAH occurs in about 10% of patients, associated with antiphospholipid (APLA) and anti-endothelial cell antibody positivity, presence of NVC alterations and RP, but generally without significant ILD [53]. Compared to SSc, MCTD-PAH seems to debut at a younger age and have a comparable 1-year survival [54].

A division into three subsets of MCTD patients was proposed: subgroup 1 with vascular damage, mainly characterized by PAH, RP and NVC positivity, subgroup 2 with ILD and myositis and subgroup 3 (with a better prognosis) featuring arthritis, articular erosions and ACPA positivity similar to RA [55].

Unfortunately, evidence regarding the role of PFTs in literature is limited. FVC and TLC proved to be stable in MCTD over ten years, while DL_CO_ showed a reduction [56]. DL_CO_ also correlated with active ILD [57], confirming its superior sensitivity in detecting ILD. Considering that PAH and ILD in MCTD and SSC are likely to have a similar pathogenic mechanism, a reasonable guide for the follow up of these patients could be derived from what is suggested for SSc.

### Idiopathic inflammatory myositis

The family of Idiopathic Inflammatory Myositis (IIMs) was traditionally used to categorize a group of autoimmune diseases characterized by muscle weakness caused by damage to skeletal muscle. The family are considered PM, DM, Necrotizing Autoimmune Myositis and Inclusion Body Myositis [58]. Except for the latter two diseases, these conditions are widely associated with visceral involvement, mainly of the skin and lung which may influence the prognosis. This classification is still under debate, with the aim of considering antibodies positivity and including other conditions such as Antisynthetase Syndrome (AS) [59]. This syndrome is characterized by the presence of the classic triad of myositis, ILD and inflammatory arthritis, in which RP, mechanic hands and unexplained fever may occur [60]. The incidence of PM and DM is calculated at about 6–7 per million [61] but is probably significantly underestimated based on the clinical presentation of these patients. Frequently, lung involvement is the unique clinical manifestation at disease onset, but during follow up the majority of these patients will develop the other clinical manifestations of the disease [62, 63]. Moreover, Sontheimer identified clinical subsets of patients without significant muscle involvement but the presence of typical skin involvement (Gottron’s papules and sign, mechanic hands, heliotrope rash) and the possibility of developing an ILD with a worse prognosis than DM [64, 65]. This condition is named Clinically Amyopathic Dermatomyositis (CADM). At present, validated criteria for AS are not available. However, several patients classified as Interstitial Pneumonia with Autoimmune Features (IPAF) for an ILD associated with Anti tRNA Synthetase Antibodies (ATSA), should be classified for both IPAF or AS depending on the referral centre [66, 67].

ILD affects about 65% of PM/DM patients, in a clinically relevant subset in 17–36% of patients, and proved to be a cause of death in about 50% of patients, especially in a subset of rapidly progressive ILD with positivity for MDA5 antibodies [67, 68]. The most common HRCT pattern is NSIP, but often an Organising Pneumonia (OP), alone or an overlap NSIP-OP, can be found [69]. AS showed similar HRCT patterns, but at higher prevalence, with up to 90% of patients with ILD [70, 71]. AS-ILD prognosis is related to specific ATSA positivity: anti Jo1 positive patients generally have myositis and arthritis with better outcome in ILD, while PL7 and PL12 positive patients showed a worse prognosis due to rapidly progressive ILD [72].

Few studies in the literature have investigated the role of PAH in IIMs. It was found in 16% of PM patients, mainly associated with Pericardial Effusion (PE) and diffuse ILD [73]. However, a large cohort of PAH patients showed IIMs in only 34 out of 5,223 patients, of which there were only 3 without significant ILD [74]. These patients had DM, and PAH was associated with SSA/Ro antibody positivity and, similar to SSc and MCTD, with skin involvement and peripheral microangiopathy. Finally, in AS a PAH was demonstrated in 16 out of 201 patients, generally associated with severe ILD. AS-PAH patients are more likely to have polyarthralgia and longstanding disease. The authors reported a 3-year survival rate of 58% but they did not find any correlation with ATSA positivity [75].

Sporadically both a restrictive disease and PAH in IIMs could be found, mainly due to muscle weakness, secondary to respiratory or cardiac muscle involvement. Several case reports are reported in literature.

Regarding PFTs, an FVC% < 60 in IIMs was correlated with a worse prognosis [76], together with Hamann Rich presentation, CADM, older age and acute/subacute onset of ILD [77]. FVC seems to be able to predict the response to therapy: in one small-scale study the decrease in the extent of ground glass opacities correlated inversely with changes in FVC [78]. FVC and DL_CO_ showed also correlation at baseline with disease extent on HRCT (with major sensitivity for DL_CO_) [79]. This data was confirmed with the CALIPER software, but after a 1-year follow up, a significant correlation was found only for TLC [80].

### SJÖGREN’S

Sjӧgren’s Syndrome (SjS) is a chronic autoimmune disease involving about 0.06% of the population characterized mainly by Sicca syndrome, due to damage to the lacrimal and salivary glands [81]. SjS pathogenesis is not completely understood yet, but B cells and a T helper type 1 and 17 responses play a key role, explaining the potential systemic involvement (lung involvement, vasculitis and lymphomagenesis) [82].

The most common SjS lung involvement is an obstructive disease with bronchial hyperresponsiveness (42–60% of patients) often insensitive to inhaled corticosteroids, bronchiolitis (12–24% of patients) and BR (7–54% of patients) [83]. The mechanism underlying these conditions seems to be local dryness in the upper respiratory tract, alteration in mucociliary clearance and the presence of hyperplastic lymphoid follicles with reactive germinal centres similar to those present in salivary glands. This impaired muco-ciliary clearance can expose patients to infective pneumonia, reported in 10–35% of patients [83].

ILD is present in 9–20% of patients. The most common HRCT pattern is NSIP (45%) followed by UIP, Lymphocitic Interstitial Pneumonia and OP (16, 15, 7% respectively, other conditions in 17%) [84].

ILD in SjS is generally stable [85], while it can be accelerated when associated with hypergammaglobulinemia, lymphopenia, reduction of FVC and positivity for Rheumatoid Factor (RF), SSa/Ro and SSb/La with low sensitivity but high specificity [86].

PAH is rarely reported in literature for SjS with some case reports. Despite these, echocardiographic elements suggestive of PAH are reported in about 25% of patients [87]. PAH is a severe condition, but, unlike other conditions, can benefit from immunosuppressant therapy [88]. PAH was also associated in SjS patients with the presence of RP, PE, hepatic injury and high titre of RF [89].

In summary, both PAH and ILD showed contradictory data. A possible explanation could be the evidence that both these conditions can have their onset years before the complete manifestation of SjS [83, 89]. Moreover, it is possible that patients with severe lung involvement that could have an impact on their prognosis, tend to be referred to a specialist perceived to be more relevant to their condition, bypassing the rheumatologist.

Regarding PFTs, few data are reported in the literature. A common finding is the reduction of Vital Capacity (VC), Forced Expiratory Volume at 1 s (FEV_1_), FEV_1_/VC ratio and DL_CO_, signalling an obstructive disease, even in non-smoking patients [90]. Obviously, an overlapping restrictive disease should be taken into account. During ILD, good correlation was found at baseline between DL_CO_ and TLC and the scores of ground-glass attenuations [91].

### Systemic lupus erythematosus

SLE is a chronic autoimmune disease potentially able to cause a systemic involvement (skin, kidney, blood, central and peripheral nervous system, etc) characterized by a deposition of immune complexes leading to organ damage [92]. Although lung involvement is not considered separately in SLE classification criteria [93], the disease is able to damage the whole respiratory tract at each level [94]. Upper airway involvement includes nasal ulcers (considered in SLE classification criteria), mucosal inflammation and sporadically severe airway obstruction with respiratory failure [94]. The most common involvement is pleurisy (also considered in SLE criteria), that can involve up to 60% of patients [95]. Another extra-parenchymal manifestation of SLE is Shrinking Lung Syndrome (SLS), a rare complication characterized by a restrictive disease without parenchymal involvement due to diaphragmatic dysfunction. This condition could affect up to 10% of patients [96]. ILD affects about 15% of SLE patients, generally with NSIP pattern [97]. SLE-ILD patients often showed a better prognosis than that seen in idiopathic forms or other CTDs. Severe parenchymal manifestations are Acute Lupus Pneumonitis (ALP) and Diffuse Alveolar Damage (DAD). The first is an acute form that involves about 1–4% of patients with an active flare of the disease and SSa/Ro positivity [98] that is hard to distinguish from infective pneumonia. DAD is a life-threatening complication that affects about 2% of patients and is characterized by damage to the alveolar-capillary membrane, generally associated with positivity for antiphospholipid antibodies (APLA) [99].

SLE is also the second autoimmune cause of PAH, with a prevalence of 0.6–17% of patients [100]. Multiple pathogenic pathways can be implicated in pathogenesis (vasculitis, pro-thrombotic state secondary to the presence of APLA, cardiac and valvular dysfunction, ILD and SLS). Therefore, SLE-PAH can be potentially classified in each PAH group, but increasing evidence suggests the utility of immunosuppressive therapy (jointly with vasodilators) for its management [94, 100].

Regarding PFTs, little evidence is available, probably due to the fact that severe lung involvement is rare and usually acute, while a chronic disease generally shows a slow decline and a good prognosis.

A reduction in DL_CO_ is reported in about 38% of asymptomatic SLE patients, but after a follow up period of 9 years none of these patients developed a lung disease [101], therefore this reduction seems not to be useful in predicting future lung involvement in SLE patients. A reduction in Maximum Voluntary Ventilation without a significant impairment in FEV_1_ and FVC was also noted in SLE patients, leading to the conclusion that fatigue, a common symptom in the disease, could also involve the respiratory muscles [102]. Regarding 6MWT, a Heart Rate Recovery (difference between Heart rate at the end of 6MWT and after 1,2 and 3 min of rest) < 16 was associated with a worse prognosis in patients with CTD-PAH (group composed of patients with lSSc, dSSc, MCTD, SLE) [103].

### Other conditions

The prevalence of lung involvement in UCTD has not been adequately studied. The major problem is a lack of consensus on classification criteria for UCTD and possible selection bias. Indeed, UCTD can represent an incomplete form or an early onset of CTD and it is reasonable to suppose that individual patients are usually referred to a specialist in the specific organs involved. In 2015, classification criteria for Interstitial Pneumonia with Autoimmune Features (IPAF) were proposed, in order to recruit these forms with ILD involvement [104]. Currently no prospective data are available in literature, but several retrospective studies report a heterogeneous population [66]. An improvement of these criteria could be useful for the earlier recruitment of CTD-ILD, in order to exploit a window of opportunity before the development of a structural damage.

An interesting speculation can also be made regarding Antiphospholipid Syndrome (APS). This is an autoimmune disease characterized by the presence of venous/arterial thrombosis or obstetrical morbidity associated to the positivity for APLA and Lupus Anticoagulant (LAC). APS often overlaps with CTDs (mainly SLE) and is associated with PAH and pulmonary embolism [105]. As already mentioned, the presence of APL can predict PAH in SLE [99]. Sporadically, ILD in APS was reported. Conversely, it should be noted that APLA are frequently discovered in IPF patients, and a LAC positivity was found in about 20% of patients [106]. Moreover, these patients frequently had vascular thrombosis, therefore satisfying classification criteria for APS [107]. The percentage of LAC positivity in IPF patients appears to be very high, considering the incidence of LAC positivity in the general population (1–5%, [108]) and in SLE (34.1%, [109]), potentially explaining the pro-thrombotic state evidenced in the disease [110].

## Conclusions

The evolution of CTD-ILD varies significantly in each patient: some identified risk factors for diagnosis and prognosis are reported in Table 1. Considering the different pathogenic pathways and manifestations of lung involvement in CTDs, a translation of the PFTs indications and their significance from IPF to CTDs should be made with caution. Table 2 summarizes the PTFs’ significance in CTDs.

**Table 1:** Risk factors for Chronic Pulmonary Disease in CTDs

DISEASE	ILD	OLD	PAH
RA	Older age; male sex, smoking history; ACPA positivity, LDH, longstanding or persistently active disease; rheumatoid nodules, articular erosions, genetic predisposition (HLA linked)	Smoking, older age	
SSDs	Scl70, Th/To positivity, dSSc, smoking, older age, rapidly progressive disease.		ACA, LAC, and anti RNA polymerase I, II, III, AECA positivity, older age, RP, NVC positivity; telangiectasias, seric NT-pro-BNP and urate, RAD, RAA, TV, PE
IIMs	Cutaneous manifestations; telangiectasias, RP, ATSA positivity (rapidly progressive for non-Jo1 positivity); older age, acute/subacute onset, CADM, Hamann Rich presentation correlated with worse prognosis		Cutaneous manifestations, peripheral microangiopathy, positivity for SSA/Ro, severe ILD, polyarthralgia, longstanding disease
SjS	Hypergammaglobulinemia, lymphopenia, RF, SSA/Ro, SSB/La positivity	Sicca syndrome, smoking	RP, PE, hepatic injury, RF positivity
SLE			APLA, cardiac or vascular dysfunction, ILD, SLS

*ACA* anticentromeric antibodies, *ACPA* Anti Citrullined Peptides Antibody, AECA Anti Endothelial Cell Antibody, *ATSA* Anti tRNA Synthetase Antibodies, *CTDs* Connective Tissue Diseases, *dSSC* diffuse Systemic Sclerosis, *HLA* Human Lymphocitic Antigen, *IIMs* Idiopathic Inflammatory Myopathies, *ILD* interstitial Lung Disease, *LAC* Lupus Anticoagulant, *LDH* Lactic Dehydrogenase, *OLD* Obstructive Lung Disease, *PAH* Pulmonary Artery Hypertension, *PE* pericardial Effusion, *RA* Rheumatoid Arthritis, *RAA* Right Atrium Area in echocardiography, *RAD* Right Axis deviation in electrocardiogram, *RF* Rheumatoid Factor, *RP* Raynaud Phenomenon, *SLE* Systemic Lupus Erythematosus, *SjS* Sjӧgren Syndrome, *SSDs* Scleroderma Spectrum Disorders, *TV* tricuspid velocity in echocardiography, *SLD* Shrinking Lung Syndrome

**Table 2:** Significance of PFTs in Chronic Lung involvement due to CTDs

Disease	Items	Correlation
RA		
ILD	Decline of FVC ≥ 10% from 68.7 or any time from baseline Decline of DL_CO_% ≥ 10% from 48.1 or any time from baseline	Mortality
	DL_CO_% ≤ 54 FVC decline ≥10% DL_CO_ decline ≥15%	Progression
SSDs		
ILD	Decline of FVC ≥10% or comprised between 5 and 10% with a decline of DL_CO_≥15%	Progression
	DL_CO_ < 80	Clinical and NVC DA
PAH	DL_CO_ ≤55	diagnosis
	FVC/DL_CO_> 1.6	diagnosis
IIMs		
ILD	FVC ≤ 60	prognosis
	TLC	Disease extent in FU
SjS		
OLD	Reduction of VC, FEV_1_, FEV_1_/VC, DL_CO_	Diagnosis and prognosis
ILD	DL_CO_, TLC	Correlation with disease extent
SLE		
PAH	HRR < 16	prognosis

*DA* Disease Activity, *DLCO* diffusing capacity for carbon monoxide, *FEV1* Forced Expiratory Volume in 1 s, *FVC* forced vital Capacity, *HRR* heart Rate Recovery, *IIMs* Idiopathic Inflammatory Myopathies, *ILD* Interstitial Lung Disease, *NVC* Nailfold Videocapillaroscopy, *PAH* Pulmonary Artery Hypertension, *PFT* Pulmonary Function Test, *RA* Rheumatoid Arthritis, *SjS* Sjögren Syndrome, *SLE* Systemic Lupus Erythematosus, *SSDs* Scleroderma Spectrum Disorders, *TLC* Total Lung Capacity, *VC* Vital Capacity

The high incidence of ILD in CTD suggest that PFTs, jointly with physical examinations, should be performed in all new diagnoses and repeated periodically regardless of referred symptoms. In RA, PFTs can be useful for highlighting an asymptomatic ILD before its clinical onset [3], and it could be useful to tailor appropriate immunosuppressive drugs. Similar data were reported for SSc [2], but not for SLE [101]. In patients with SSDs and IIMs, the frequency and the severity of chronic lung involvement suggest that it would be wise to perform an HRCT jointly with PFTs in all new patients, but PFT items appear to be less accurate than in IPF in the description of lung function during the course of the disease or in response to treatment. Considering this, a composite index could be more useful than the evaluation of a single item.

In view of this, more exhaustive studies regarding risk factors together with PTFs could be useful for early diagnosis and treatment before the stabilization of a structural damage, improving both patient survival and quality of life.
